# The impact of exercise intensity on depression in fibromyalgia: a randomized controlled trial

**DOI:** 10.3389/fpsyg.2024.1400590

**Published:** 2024-09-27

**Authors:** Guilherme Torres Vilarino, Danilo Reis Coimbra, Henrique Pereira Neiva, Alexandro Andrade

**Affiliations:** ^1^Department of Physical Education, Health and Sports Science Center (CEFID), Santa Catarina State University (UDESC), Florianopolis, Brazil; ^2^Faculty of Physical Education and Sport, Life Sciences Institute, ICV, Federal University of Juiz de Fora, Governador Valadares, MG, Brazil; ^3^Department of Sport Sciences, University of Beira Interior, Covilhã, Portugal; ^4^Research Center in Sports Sciences, Health Sciences and Human Development, CIDESD, Covilhã, Portugal

**Keywords:** affective disorders, depressive symptoms, mental health, strength training, resistance training, physical activity

## Abstract

**Background:**

Fibromyalgia (FM) is characterized by widespread chronic pain. Although pain is the main symptom, approximately 90% of patients have depression. This study aimed to analyze the effects of Resistance Training (RT) with low and high intensity on depression in patients with FM.

**Methods:**

Thirty-eight women with FM and 31 healthy women were allocated to the low-intensity, high-intensity, preferred-intensity, and control groups. The patients underwent 8 weeks of supervised RT, with two sessions per week of approximately 1 h. The low-intensity resistance training group (LIRT) performed two sets of 12 maximum repetitions. The high-intensity resistance training group (HIRT) performed four sets with six maximum repetitions, and the preferred intensity group (PI) performed three sets, with eight to 12 repetitions, according to the patient’s tolerance. The healthy control group did not perform any type of exercise. Depression was assessed using the Beck depression inventory before the start of the intervention, after 4 and 8 weeks.

**Results:**

FM patients have higher levels of depression than women without the disease. After 4 weeks, there was a difference in depressive symptoms between the HIRT and LIRT (*p* = 0.048), and the PI and LIRT (*p* = 0.048).

**Conclusion:**

Prescribing RT with low or high intensity did not significantly reduce depression in patients with FM after 8 weeks, however, analyses between groups after 4 weeks indicated that low-intensity training is more effective than high-intensity training. The prescription of RT exercise to FM could vary among low, high, and preferred intensity, following the patient’s tolerance for pain.

**Clinical trial registration:**

https://ensaiosclinicos.gov.br/rg/RBR-74pcmw, RBR-74pcmw.

## 1 Introduction

Fibromyalgia (FM) is a syndrome characterized by widespread chronic pain, that is more common in women (Häuser et al., 2015; Andrade et al., 2018b, 2020b; Gendreau et al., 2024). The syndrome has a high prevalence, and it is estimated that approximately 2.7% of the world population has the diagnosis, ranging from 2.1 to 6.4%, according to the country analyzed (Branco et al., 2016). Although pain is the main symptom, there is great deterioration in the patient’s mental health, and 90% of affected patients present mood disorders, such as depression and excessive anxiety (Andrade et al., 2017). Among these, depression is the most widely studied and has an important relationship with other symptoms (Vilarino et al., 2021). Depression is a common psychiatric disorder in 4.4% of the world’s population (WHO, 2017) and the prevalence increases in people with chronic pain (Gerrits et al., 2014). In FM patients, studies have shown comorbidity with depression, however, there is still no conclusion about which of the disorders leads to the other. Chang et al. (2015) showed that patients with FM are 7.46 times more likely to develop depression than patients without the syndrome and that people with depression are 6.28 times more likely to develop FM, thus a bidirectional relationship between disorders is observed. Another important issue is the risk of suicide that accompanies FM patients who present depressive symptoms (Varallo et al., 2024). This fact demonstrates the importance of this topic.

Due to the difficulties in establishing the diagnosis and the different hypotheses regarding the cause, FM patients face many challenges in the course of treatment (Bellato et al., 2012), which mainly consists of the use of medications, such as analgesics and antidepressants (Macfarlane et al., 2017). However, the continued use of some medications has undesirable side effects (Häuser et al., 2010), in addition to high financial expenses for patients (Lacasse et al., 2016). Thus, other treatment possibilities have been studied that involve fewer financial resources, do not have side effects, and are effective in physical symptoms and mental health (Sieczkowska et al., 2019, 2020a; Albuquerque et al., 2022; Vilarino, 2024). Among the treatment alternatives, physical exercise (PE) has been widely recommended, having the ability to reduce depression in the general population (Cooney et al., 2013; Schuch et al., 2016) and in patients with other diseases, such as cancer (Chen et al., 2014), Lupus erythematosus (O’Dwyer et al., 2017) and FM (Andrade et al., 2017; Vilarino et al., 2021). One modality of PE that has been studied in recent years as an alternative treatment is resistance training (RT), however, most of the reported results are about physical symptoms like pain, strength, and fatigue (Vilarino et al., 2021, 2022a; Albuquerque et al., 2022; Bastos et al., 2023). Nonetheless, systematic reviews conducted by Andrade et al. (2018a) and Vilarino et al. (2021) found that RT reduces depression and can be used as a strategy in the management of this symptom.

Despite the positive results of RT in the depression of patients with FM, some questions regarding the intervention need to be further investigated (Vilarino et al., 2021; Albuquerque et al., 2023). Although RT significantly reduced depressive symptoms independent of total prescribed volume (intervention length × frequency × session duration), studies with patients with FM that compare the optimal dosage to achieve significant clinical improvements have not yet been reported. The intensity of the RT could lead to different responses, allowing greater variety in the training prescription. Different exercise intensities could induce a hypoalgesic or hyperalgesic response, leading patients with FM to decrease adherence, experience adverse events (like pain), and drop out of RT programs. Häkkinen et al. (2001) investigated the effects of 21 weeks of progressive RT on neuromuscular function and subjectively perceived symptoms in premenopausal women with FM. The RT protocol started with 15–20 repetitions (40–60% of 1 RM) in the first 3 weeks. Between the fourth and seventh weeks, the load was increased to 60–70% of 1 RM (10–12 repetitions) and during the eighth and 14^th^ weeks, the number of repetitions was 8–12 (60–80% of 1RM). Finally, in the final 7 weeks (15–21), 5–10 repetitions were performed with loads of 70–80% of 1 RM. The authors concluded that progressive RT can safely be used in the treatment of FM, and a significant decrease in depression was observed, however, there were no comparisons between the intensity performed by the patients with FM.

Studies evaluating the different effects of low and high-intensity RT on the mental health of patients with FM are essential. However, to date, most studies have evaluated the effect of aerobic exercise (AE) (Cooney et al., 2014; Stanton and Reaburn, 2014). In this sense, da Cunha Ribeiro et al. (2018) compared the effects of different RT models (self-selected or prescribed intensity), on pain in FM patients. Although neither RT model reduced pain in FM patients, the self-selected load was performed with a free number of repetitions until achieving a rating of perceived exertion score of 7 (i.e., very hard). Since FM patients present depression, which is related to other symptoms, such as pain, investigating the effect of low and high-intensity RT could offer practical solutions in the treatment of these patients. Thus, the aim of the current study is to analyze the effects of a low, high, and preferred intensity RT protocol on depression in patients with FM. Our hypothesis was that both low-intensity and high-intensity training, as well as preferred intensity, would show similar results in improving depressive symptoms.

## 2 Materials and methods

### 2.1 Design

This is a controlled, randomized, blinded clinical trial developed at the Santa Catarina State University, at the Center for Health and Sports Sciences, which followed the guidelines of the Consolidated Standards of Reporting Trials (CONSORT) (Schulz et al., 2010). The study was carried out from August 2019 to February 2020, conducted in accordance with the ethical standards required by the Declaration of Helsinki and Resolution 466/12 of the National Health Council of Brazil, and was approved by the Research Ethics Committee of UDESC (CAAE 24584213.0.0000.0118). The research is registered in the Brazilian Registry of Clinical Trials (no. RBR-74pcmw).

### 2.2 Participants

The study included 31 healthy control women and 38 FM patients with experience in RT (28 randomized and 10 from the database of the extension project). The study participants were recruited from the project “Sport and exercise psychology applied to Health,” developed by the Santa Catarina State University. This program receives patients from hospitals and Basic Health Units in a city in southern Brazil. All participants with FM were diagnosed according to the criteria established by the American College of Rheumatology (Wolfe et al., 2016).

The inclusion criteria for patients with FM were (1) medical diagnosis of FM, (2) female, (3) aged between 18 and 70 years, and (4) a medical certificate providing clearance for the practice of PE. The exclusion criteria were (1) patients with cardiac, pulmonary, muscle, or joint problems that prevented them from participating in exercise sessions, (2) pregnant or lactating women, (3) a history of PE practice in the previous 3 months (exercised at least twice a week, lasting at least 30 min), (4) perform another type of physical exercise while participating in the study, and (5) an attendance frequency of less than 75% in training sessions. For the control group with healthy women, the following inclusion criteria were adopted (1) women aged between 18 and 70 years, (2) healthy (without regular medical treatment for any disease), and (3) without complaints of pain, joint stiffness, and sleep disorders.

The sample size was calculated based on the depression variable, verified by the BDI, through the analysis of the standard deviation of the study published by Gavi et al. (2014) The G*Power 3.1 (Faul et al., 2007) program was used to determine the sample size. Considering an alpha risk of 0.05 and power of 0.95, a total sample of at least 56 participants was required to be allocated to the four groups.

### 2.3 Procedures

**Figure 1:**
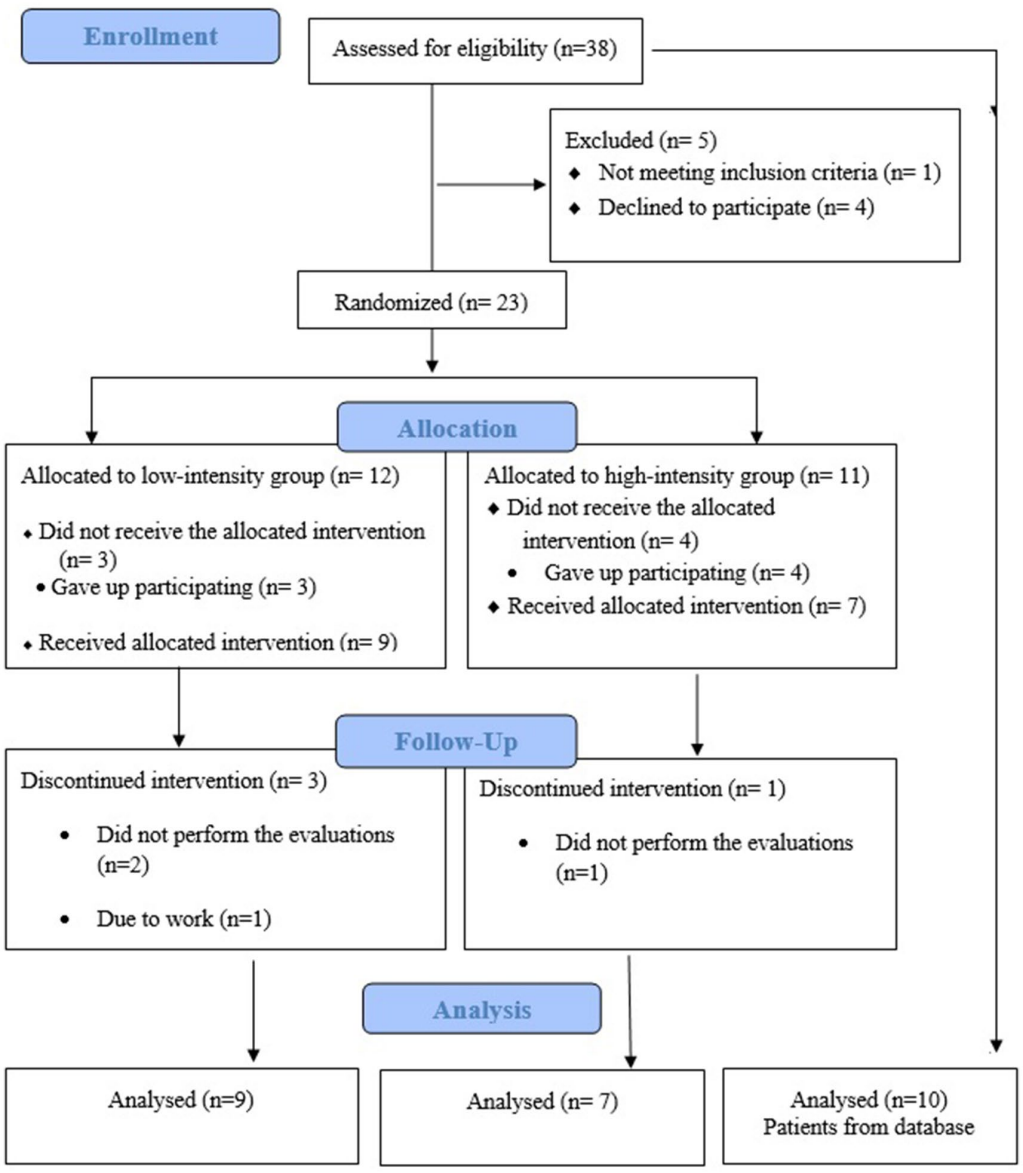
Flow diagram of patient recruitment following CONSORT guidelines Intervention.

After the initial contact, individual interviews were scheduled with the participants to explain in detail the research procedures, evaluations, and interventions. The participants answered questionnaires about sociodemographic and clinical characteristics and the Beck Depression Inventory (BDI) (Beck et al., 1961). After data collection, participants were allocated to one of two intervention groups: Low-Intensity Resistance Training Group (LIRT) or High-Intensity Resistance Training Group (HIRT), randomized using a 1: 1 computer-generated allocation.^1^ The patients did not know which group they had been allocated to. The participants in the Healthy Control Group (HC) and patients in the Preferred Intensity Group (PI), were not randomized. The inclusion flowchart for LIRT and HIRT participants is shown in Figure 1. To avoid any circadian effect on symptoms, the exercise sessions and assessments took place at the same time of day. Written informed consent was obtained from all participants.

The patients underwent 8 weeks of supervised RT, with two sessions per week. Each training session lasted from 45 min to 1 h. All participants were beginner level and performed two familiarization sessions before starting the RT program, during which they were taught to perform the exercises. In the three intervention groups, the exercise protocol was the same, however, the load was individualized. Initially, the patients performed a warm-up, including some exercises with a lower load than usual, and then performed the following exercises: bench press, low row, squat, leg press, shoulder press, and standing calf raise. The exercise protocol followed the recommendations for prescribing resistance exercises (Hass et al., 2001; Kraemer and Ratamess, 2004), and this protocol was already used in a previous study (Vilarino et al., 2022b). LIRT exercises consisted of two sets of 12 maximum repetitions (60% of 1RM to concentric failure), with a one-minute rest between sets. In the HIRT, the exercises were composed of four sets with six maximum repetitions (85% of 1RM to concentric failure), with an interval of 2 min between sets. In the PI, the exercises were performed in three sets, with 8–12 repetitions, according to the self-selected load based on the patient’s subjective effort, with an interval between sets of 1 min. The exercise progression was carried out once a week when the patient’s adaptation was observed. The load was adjusted when the participant was able to perform more repetitions than recommended for their group with an adequate movement pattern. The increase in load varied concerning the exercise used; when the exercise was performed on a machine, such as in the low row, one tablet was increased. The increase in leg exercises (Squats and leg press) was five kilos. In the other exercises, the increase was two kilos. All training sessions were conducted in groups of 4–5 women in the university’s gym and supervised by a professional instructor in RT training. No motivational strategy was applied in any group. The adherence rate to the exercise protocol was calculated as the ratio between the number of exercise sessions performed and the number of sessions prescribed.

Women in the healthy control group were asked not to change their daily activities. Groups received guidance not to perform home exercise programs.

### 2.4 Outcome measures

The sociodemographic and clinical characteristics of the patients (age, educational level, time of diagnosis, and main symptoms) were obtained through a standardized interview.

The Beck Depression Inventory (BDI) was used to assess symptoms of depression. This tool is used worldwide and was developed by Beck et al. (1961) and validated in Brazil by Gorenstein and Andrade (1996). The BDI can be self-applied and consists of 21 items on depressive symptoms, referring to the previous 7 days, with a final score ranging from 0 to 63. The questionnaire addresses items that refer to sadness, pessimism, feelings of failure, lack of satisfaction, feelings of guilt, feelings of punishment, self-deprecation, self-accusations, suicidal ideas, crying crises, irritability, social withdrawal, indecision, body image distortion, inhibition to work, sleep disorder, fatigue, loss of appetite, weight loss, somatic concern, and decreased libido. The scale was categorized as: no depression or minimal depression (0–9), mild to moderate depression (10–18), moderate to severe depression (19–29), and severe depression (30–63) (Gorenstein and Andrade, 1998). This tool has shown a good ICC coefficient for retest reliability (ICC = 0.89; 95%CI 0.82–0.93) and a Cronbach’s alpha coefficient of 0.93.

**Figure 2:**
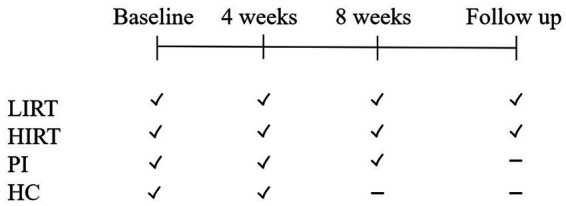
Assessment periods of depression in the groups. ✓ = evaluation performed; ▬ = no evaluation was carried out; LIRT = Low intensity resistance training; HIRT = High Intensity resistance training; PI = Preferred Intensity; HC = Healthy control.

The BDI assessments were performed by researchers previously trained and blinded to patient allocation. The participants in the LIRT and HIRT groups were reassessed after 4 weeks and 8 weeks of training, and 4 weeks after training cessation. Data were evaluated for the PI referring to the initial evaluations, and four and 8 weeks of intervention, and the HC was evaluated for a period of 4 weeks (Figure 2).

### 2.5 Statistical analysis

Exploratory data analysis was performed to verify the dispersion of data (Kolmogorov–Smirnov test and dispersion graph). Due to the variability between subjects, Generalized Linear Mixed Models were performed for the dependent variable (depression) with Gamma regression. Gamma regression uses a Gamma distribution with a log link, which should be used when the dependent variable contains all positive values and is skewed toward larger values. Experimental condition (Low Intensity, High Intensity, Preferred Intensity, and Healthy Control) and time (baseline, 4 weeks, 8 weeks, and follow-up), were adopted as fixed effects, and subjects as a random effect. The Bonferroni test was performed to identify the differences between groups, time, and the interactions (group*time). The type of repeated covariance was based on the AR matrix (1) and the criterion for the model was the Akaike information criterion (AIC). To validate the adequacy of the model, the normality of the residues was also analyzed by the Q-Q Plot.

Hedge’s g (g) (x ®1-x ®2/SD Pooled) was calculated to assess the magnitude of significant differences between the pre-and post-intervention and between groups. The effect size (ES) was interpreted as follows: <0.2 without effect; 0.2–0.4 small; 0.5–0.7 moderate; ≥0.8 large (Cohen, 2013).

For all analyses, a significance level of *p* < 0.05 was adopted. Data are presented as mean ± standard deviation and 95% Confidence Interval. Intention-to-treat analysis (ITT) was performed. The Statistical Software Statistical Package for the Social Science (SPSS) version 20.0 (IBM Corp., Armonk, NY, United States) was used for all statistical tests.

## 3 Results

During the intervention period, no adverse events were reported, and the final sample was composed of 26 patients with FM [randomized to LIRT (*n* = 9), HIRT (*n* = 7), and from databased (*n* = 10)], and 31 healthy women. The average age of the participants was 50.85 ± 11.47 years. Most FM patients from the LIRT and HIRT groups were employed during the study period and had been diagnosed for more than 60 months. It was observed that more than 75% of the patients with FM reported tiredness, localized pain, non-restorative sleep, and joint stiffness, while the participants of the HC did not report major complaints (45.5% tiredness). Regarding the use of antidepressants, 65.4% of FM patients reported their use, and only 12.1% of HC women. Table 1 presents the sociodemographic and clinical characteristics of the participants in each group. Adherence rates in the LIRT and HIRT were 77.7, and 85.71%, respectively. RT adherence data were controlled by the instructor in each session. In this sense, the RT was delivered as planned.

**Table 1:** Sociodemographic and clinical characteristics of the participants in each group.

	LIRT (*n* = 9)	HIRT (*n* = 7)	PI (*n* = 10)	HC (*n* = 31)	*p*
Age (year)	57.8 ± 9.9	46.2 ± 8.3	57 ± 9.0	47.8 ± 11.8	0.02^*^
**Marital status (%)**					
With partner	5 (55.6)	4 (57.1)	4 (40)	21 (63.6)	0.62
Without a partner	4 (44.4)	3 (42.9)	6 (60)	12 (36.4)	
**Have an occupation? (%)**					
Yes	0 (0)	3 (42.9)	3 (30)	13 (39.4)	0.10
No/away/retired	9 (100)	7 (57.1)	7 (70)	20 (60.6)	
**FM diagnosis time (months)**					
1–24 months	1 (11.1)	2 (28.6)	0 (0)	–	0.32
25–60 months	2 (22.2)	0 (0)	1 (10)	–	
More than 60 months	6 (66.7)	5 (71.4)	9 (90)	–	
**Most common symptoms (%)**					
Tiredness	7 (77.8)	7 (100)	9 (90)	15 (45.5)	<0.01^#^
Fatigue	5 (55.6)	7 (100)	8 (80)	4 (12.1)	<0.01^#^
Localized pain	8 (88.9)	6 (85.7)	10 (100)	7 (21.2)	<0.01^#^
Generalized pain	6 (66.7)	7 (100)	9 (90)	0 (100)	<0.01^#^
Non-restorative sleep	7 (77.8)	7 (100)	8 (80)	11 (34.4)	<0.01^#^
Memory failure	5 (55.6)	6 (85.7)	6 (60)	8 (24.2)	<0.01^#^
Joint stiffness	7 (77.8)	7 (100)	10 (100)	4 (12.1)	<0.01^#^
Excessive anxiety	7 (77.8)	4 (57.1)	7 (70)	6 (18.2)	<0.01^#^
**Use of antidepressants**					
Yes	7 (77.8)	4 (57.1)	6 (60)	4 (12.1)	<0.01^#^
No	2 (22.2)	3 (42.9)	4 (40)	29 (87.9)	

*No significant difference was found after *post hoc* analysis (Bonferroni); ^#^ HC vs. LIRT, HIRT, and PI.

When the mean value of depression at baseline was compared between working and non-working patients, no significant difference was observed (*p* = 0.88). Considering the number of symptoms (tiredness, fatigue, localized pain, non-restorative sleep, memory failure, joint stiffness, excessive anxiety), those who reported more than seven symptoms showed a higher mean depression value (18.95 ± 10.63 versus 6.25 ± 1.50; *p* = 0.02; g = “large”).

**Figure 3:**
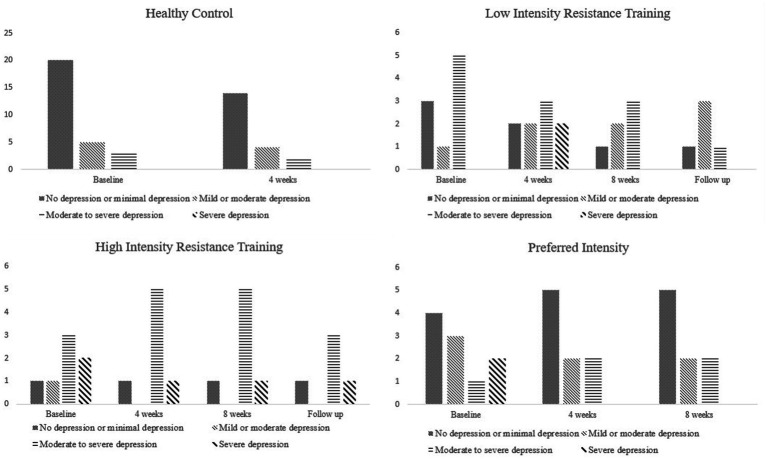
Classification of the level of depression by group during the periods analyzed.

The analysis of the classification of depression levels by the BDI at baseline showed that the PI had a greater number of patients with FM with no depression or minimal depression, compared to the LIRT and HIRT. The PI presented a greater number of patients with minimal depression over the intervention period. At baseline, the LIRT and HIRT groups of patients with FM included patients with severe depression, however, at the end of the intervention, only the HIRT presented patients with this classification. Most of the HC participants presented the classification of no depression to minimal or mild to moderate depression (Figure 3).

In the Generalized Linear Mixed Modeling, independent variables (group condition: Low Intensity, High Intensity, Preferred Intensity, and Healthy Control), and time (baseline, 4 weeks, 8 weeks, and follow-up) were adopted as fixed variables, and subjects as a random effect. The group presented a significant effect (*F*_3, 113_ = 3.458; *p* = 0.19). However, no significant effect was found on time (*F*_3, 113_ = 2.177; *p* = 0.95) or in the interaction group x time (*F*_6, 113_ = 1.076; *p* = 0.381). The random effect was significant (*β* = 2.628; SE = 0.287; *t* = 9.150; *p* < 0.001; 95% CI: 2.059, 3.197) for the intercept between group and time. The quality of the model fit was satisfactory (AIC = 220, 612; BIC = 228, 57). The Q-Q Plot of the residues’ normality validated the model’s adequacy. The marginal R^2^ was significally (*R*^2^ = 0.122; SE = 0.061; *p* = 0.46). Results of the Generalized Linear Mixed Modeling are presented in Table 2.

**Table 2:** Comparison of depression levels (BDI) among participants with FM submitted to different intensities of resistance training and health control.

Group	Baseline *M* ± SD (CI 95%)	4 weeks *M* ± SD (CI 95%)	8 weeks *M* ± SD (CI 95%)	Follow up *M* ± SD (CI 95%)	4 weeks – Baseline *M* ± SD (CI 95%)	*p*	8 weeks – Baseline *M* ± SD (CI 95%)	*p*	Follow-up – 8 weeks *M* ± SD (CI 95%)	*p*
LIRT (*n* = 9)	13.84 ± 3.98 (7.84; 24.46)	**16.05 ± 4.61**^**#,!**^ **(9.09; 28.35)**	16.20 ± 4.85 (9.09; 29.34)	11.56 ± 3.59 (6.25; 21.38)	2.20 ± 2.04 (−2.97; 7.38)	1.0	2.35 ± 2.76 (−4.17; 8.88)	1.0	−4.64 ± 2.66 (−11.79; 2.52)	0.507
HIRT (*n* = 7)*	20.57 ± 6.69 (10.79; 39.21)	**15.82 ± 5.23**^**#**^ **(8.21; 30.46)**	17.56 ± 5.84 (9.08; 33.95)	14.96 ± 5.08 (7.63; 29.32)	−4.75 ± 3.21 (13.38; 3.87)	0.85	−3.01 ± 3.63 (−11.54; 5.53)		−2.61 ± 2.89 (−9.94; 4.72)	1.0
PI (*n* = 10)	9.00 ± 2.45 (5.25; 15.45)	**7.18 ± 1.99**^**!**^ **(4.14; 12.45)**	8.39 ± 2.35 (4.81; 14.60)	–	−1.82 ± 1.18 (−4.68; 1.04)	0.37	−0.617 ± 1.38 (−3.34; 2.11)	0.65	–	–
HC (*n* = 31)*	7.16 ± 1.11 (5.26; 9.74)	7.18 ± 1.19 (5.17; 9.97)	–	–	0.03 ± 0.69 (−1.34; 1.39)	0.97	–	–	–	–
LIRT X HIRT	6.72 ± 7.79 (11.44; 24.89)	−0.23 ± 6.97 (−16.08; 15.61)	3.40 ± 6.22 (−15.72; 8.93)	3.40 ± 6.22 (−8.93; 15.72)	–	–	–	–	–	–
*p*	0.91	1.0	0.86	0.58	–	–	–	–	–	–
LIRT X PI	4.84 ± 4.67 (−6.51; 16.20)	8.87 ± 5.02 (−4.29; 22.03)	7.81 ± 5.39 (−5.26; 20.88)	–	–	–	–	–	–	–
*p*	0.91	0.40	0.44	–	–	–	–	–	–	–
HIRT X PI	11.57 ± 7.13 (−7.12; 30.26)	8.63 ± 5.60 (−5.31; 22.58)	9.18 ± 6.30 (−6.13; 24.48)	–	–	–	–	–	–	–
*p*	0.91	0.44	0.44	–	–	–	–	–	–	–
HC X LIRT	−6.69 ± 4.13 (−17.51; 4.13)	−8.87 ± 4.76 (−21.65; 3.92)	–	–	–	–	–	–	–	–
*p*	0.54	0.39	–	–	–	–	–	–	–	–
HC X HIRT	−13.41 ± 6.79 (−17.51; 4.13)	−8.63 ± 5.37 (−22.25; 4.99)	–	–	–	–	–	–	–	–
*p*	0.54	0.44	–	–	–	–	–	–	–	–
HC X PI	−1.84 ± 2.69 (−7.88; 4.18)	0.00 ± 2.32 (−4.60; 4.60)	–	–	–	–	–	–	–	–
*p*	0.91	1.0								

*Difference between groups (*p* < 0.05); ^#,!^ Difference between groups in 4 weeks.

**Figure 4:**
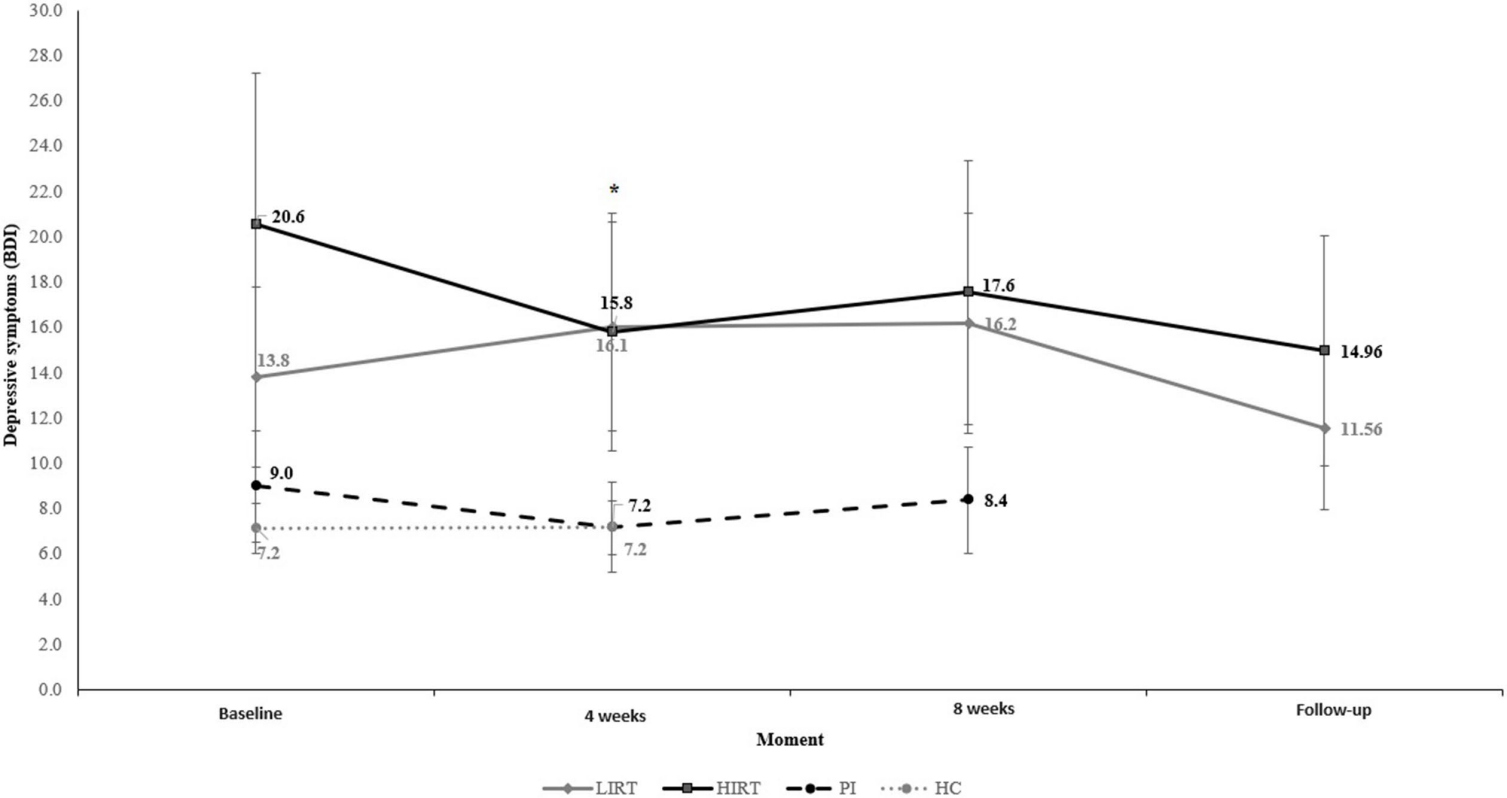
BDI values between experimental conditions over time. *p < 0.05.

Figure 4 illustrates the BDI between experimental conditions and across time. Compared with the LIRT, the HC had fewer depressive symptoms (β = −0.660; SE = 0.327; *t* = −2.020; *p* = 0.046; 95% CI: −1.307, −0.013; ES = 0.925, “large”). In the second assessment, after 4 weeks, there was a difference between the HIRT and LIRT (β = −0.411; SE = 0.205; *t* = −2.002; *p* = 0.048; 95%CI: −0.817, −0.004; ES = 0.017, “without effect”), and the PI and LIRT (β = −0.374; SE = 0.187; *t* = −1.996; *p* = 0.048; 95%CI: −0.745, −0.003; ES = 0.845, “large”) for depressive symptoms.

On the other hand, the LIRT showed a delta change in depressive symptoms in the opposite direction after 4 weeks, when compared with HIRT (Δ_LIRT_ = 2.21; Δ_HIRT_ = −4.75) and PI (Δ_LIRT_ = 2.21; Δ_PI_ = −1.82).

## 4 Discussion

The study aimed to analyze the effects of a low, high, and preferred intensity RT protocol on depression in patients with FM. After analyzing the results, it was not possible to identify differences between the groups post-intervention. The findings showed that none of the intensities of RT (low, high, and preferred) reduced depressive symptoms in patients with FM after 8 weeks of intervention.

Depression is characterized by reduced mood, loss of interest, reduced pleasure, a feeling of guilt, low self-esteem, and loss of appetite, being considered by some researchers as a symptom of FM and by others as a percussive disease (Chang et al., 2015). Therefore, it is common for patients with FM to present higher rates of depressive symptoms when compared to HC, however, in our study we observed significant differences only between HIRT and HC at the beginning of the study. This result is in line with what is pointed out in the literature, where the prevalence of depression in the general population is 10% (WHO, 2017) while in FM patients it is 90% (Andrade et al., 2017). Despite this, we observed that some participants in the HC were also using antidepressant medications, even though they were not undergoing treatment monitored by doctors during the study period. This fact is due to self-medication, which is common in Brazil (Arrais et al., 2016).

Because FM is a disabling syndrome, it is common for patients to be absent from their occupational activities (Andrade et al., 2019; D’Onghia et al., 2022). In our study, we observed that most participants were absent. However, this is partly explained by the shorter time available to participate in the study among employed patients.

Regardless of the diagnosis (depression or FM), the regular practice of PE is recommended as an alternative treatment for both groups of patients (Schuch et al., 2016; Sieczkowska et al., 2020b). Given this, several studies have analyzed different modalities of PE (Gavi et al., 2014; Macfarlane et al., 2017; Atan and Karavelioğlu, 2020), among which RT has shown positive results in improving depression in patients with FM (Andrade et al., 2017, 2018a; Vilarino et al., 2021).

In general, RT with moderate to high intensity has been used in some studies (Gavi et al., 2014; Andrade et al., 2019). In a recent study, a significant reduction in depression symptoms was observed after 4 weeks of RT (Andrade et al., 2019). However, the authors did not specify the intensity of the training. Assumpção et al. (2018) found that 12 weeks of RT improved FM symptoms, especially depression. The protocols used in both studies were similar to those performed by the PI group, with a self-selected load. However, in the present study, patients in the PI group did not present reduced depressive symptoms after 4 or 8 weeks of RT. On the other hand, we found that the classification of depression severity decreased among patients, and at the end of the study, only one participant was classified as having severe depression.

Although, to date, no experimental studies have been published analyzing the effects of low and high-intensity RT on depressive symptoms in patients with FM, some studies showed that physically active patients have lower levels of severe depression (Andrade et al., 2017), and that PE has antidepressant effects regardless of the intensity (Schuch et al., 2016). However, it was not possible to observe this antidepressant effect at the different intensities analyzed after 8 weeks.

Gordon et al. (2018), through a meta-analysis and meta-regression of an RCT, found that RT reduced depressive symptoms among adults regardless of health status. Most of the intervention was performed with low to moderate intensity (relative intensity: <80% 1RM, 12–16 RPE or authors reported exercise as low/moderate intensity). In four studies RT was performed with vigorous intensity (≥80% 1RM, >16 RPE, or authors reported exercise as vigorous intensity). In a univariate analysis, both intensities of RT were favorable to reducing depression, with no difference observed between them.

Although there were no significant differences after 4 weeks of intervention, differences between the groups were observed in this period. The delta change in the LIRT presented higher means on the BDI, while the HIRT and PI groups showed a reduction in delta changes of depressive symptoms. These results indicate a slight advantage for HIRT, since patients in this group presented lower BDI values, while LIRT patients presented higher values after the intervention. The results found contradict our initial hypothesis that both groups would present improved depression in the participants, but are in line with another recent study that found no significant differences after 4 weeks of low-and high-intensity RT in the mood states of FM patients (Vilarino et al., 2022b). In others studies, a greater effect was observed in individuals undergoing high-intensity RT. In the study conducted by (Singh et al., 2005), 61% of elderly people undergoing high-intensity training achieved a 50% reduction in depressive symptoms, while only 29% of elderly people undergoing low-intensity training achieved the same reduction.

Although the intervention period was not long, other studies with a short intervention period showed positive results (Bircan et al., 2008; Andrade et al., 2019). Nevertheless, the training frequency in those studies was higher (three times a week), suggesting that the frequency may be important for the prescription when the goal is to reduce depressive symptoms. PE promotes the release of hormones and mood-regulating substances (Murri et al., 2015), and thus a higher frequency of training could positively influence brain biochemistry, making patients feel a greater sense of well-being. In addition, it should be noted that a higher frequency of training promotes greater socialization, which is important for patients with depression (McKillop et al., 2017). Regarding the possible effects of high and low-intensity training on psychophysiological responses, which justify changes in mental health issues, they are still not well understood, given the divergences in results found. Thus, prescribing a training protocol with an emphasis on frequency rather than intensity may be an effective possibility for this population. Likewise, during training, the patient having the possibility to choose the load and perform the exercises according to their perceived effort can help in their perception of competence and self-esteem, making them feel pleasure (Andrade et al., 2020a), which can help in adherence to training.

### 4.1 Limitations and future directions

The current study has strengths and limitations. We emphasize that the number of patients evaluated in each group is small and this may have affected the results found, although the sample calculation was performed, and a sufficient number of patients were investigated. The PI group was not randomized and had no follow-up assessment, limiting our analysis. Another limitation is that patients in the PI group had more patients with minimal depression than the other intervention groups at baseline. Furthermore, there was a difference in the average age between the groups. Another issue is the use of antidepressant medications that patients continued to use during the study. Therefore, our findings need to be considered with caution and new more robust studies should be carried out, to better test the hypothesis that different physical exercise intensities can produce different clinical results.

### 4.2 Strengths, innovations and applications

Despite the limitations, the current study is the first to investigate the effects of RT with low and high intensity in patients with FM, presenting an innovative contribution to the debate regarding the prescription of PE for patients with FM, and directly assisting in practice. Additionally, no adverse events were reported by the patients in the HIRT. Therefore, it can be hypothesized that two sessions per week of high-intensity RT, supervised by an expert professional instructor is safe for patients with FM, and in the long term may present better results than LIRT, which had an increase in the BDI score (not significant).

Our study allows health professionals in fields such as medicine, physiotherapy, and physical education to consider different intensities of physical exercise, adapting the sessions to the clinical conditions of the patients but also allowing greater flexibility and intervention options.

## 5 Conclusion

We can conclude that 8 weeks of low, high, or preferred intensity of RT do not lead to different results in reducing depression in patients with FM. This suggests that the magnitude of the intensity of RT had similar effects on depressive symptoms. From a practical point of view, the prescription of RT exercise to FM could vary among low, high, and preferred intensity, in accordance with the patient’s tolerance for pain, perception of effort, and self-efficacy, as well as their safety, and supervised and monitored by a professional with expertise in RT prescription. Further studies are needed to investigate the underlying mechanisms and effects of different intensities of RT on depression in patients with FM, to understand better the best strategies for the treatment of FM syndrome.

## Data Availability

The raw data supporting the conclusions of this article will be made available by the authors, without undue reservation.

## Footnotes

^1^http://www.randomization.com
